# Psychological Effects of People Isolated in Hubei Due to COVID-19 Epidemic

**DOI:** 10.3389/fpsyt.2021.597894

**Published:** 2021-07-28

**Authors:** Jiaying Gong, Guanmao Chen, Zhangzhang Qi, Shuming Zhong, Ting Su, Youling Pan, Jurong Wang, Li Huang, Ying Wang

**Affiliations:** ^1^Medical Imaging Center, First Affiliated Hospital of Jinan University, Guangzhou, China; ^2^Department of Radiology, Six Affiliated Hospital of Sun Yat-sen University, Guangzhou, China; ^3^Institute of Molecular and Functional Imaging, Jinan University, Guangzhou, China; ^4^Department of Psychiatry, First Affiliated Hospital of Jinan University, Guangzhou, China

**Keywords:** coronavirus, epidemic, psychological effects, isolation, anxiety

## Abstract

The Coronavirus Disease 2019 (COVID-19) epidemic broke out from Wuhan in Hubei province, China, spread nationwide and then gradually developed into other countries in the world. The implementation of unprecedented strict isolation measures has affected many aspects of people's lives and posed a challenge to psychological health. To explore whether people isolated for 14 days due to having contact with COVID-19 patients had more psychosocial problems. We conducted an online survey from February 29 to March 10, 2020. Depression, anxiety, post-traumatic stress disorder (PTSD) symptoms, and coping style were assessed by the Patient Health Questionnaire-9 (PHQ-9), 7-item Generalized Anxiety Disorder Scale (GAD-7), Impact of Event Scale-Revised (IES-R), and Simplified Coping Style Questionnaire-20-Chinese Version. This study included 1,315 isolated respondents in Hubei province (58.5% located in Wuhan). 69.3% respondents isolated at home, 30.7% respondents isolated at centralized quarantined spot. Of all respondents, 66.8% reported depressive symptoms, 49.7% reported anxiety symptoms, 89.0% reported PTSD symptoms. The Cronbach α of the IES-R, PHQ-9, GAD-7, and total SCSQ-20 were 0.935, 0.847, 0.843, and 0.888, respectively. Persons who isolated at home were associated with a lower risk of PTSD, depressive and anxiety symptoms (*P* < 0.01). People who knew someone to have COVID-19 were associated with severe symptoms of PTSD symptoms (*P* = 0.001). As for coping style, higher level of passive coping style was associated with severe symptoms of PTSD, depression and anxiety (*P* < 0.001). Our findings identify that person isolated during the COVID-19 epidemic was associated with high proportion of depression, anxiety, and PTSD symptoms. Public health officials should be aware of and prepared to take necessary measures.

## Introduction

Coronavirus Disease 2019 (COVID-19), formerly known as 2019 novel coronavirus was first identified in late December 2019 in Wuhan City in China (1). From the end of December 2019, COVID-19 began to spread rapidly throughout Hubei Province and other areas in China, now it has exploded all over the world (2). As of March 18th, 2020, according to the National Health Commission (https://news.qq.com//zt2020/page/feiyan.htm), there were 179,180 people had been diagnosed with COVID-19 cases worldwide, including 81,163 in China, and 83.5% of them happened in Hubei province. So far, more than 4% infected patients had died from this new viral infection (https://news.qq.com//zt2020/page/feiyan.htm), mainly due to pneumonia and other respiratory complications such as septic shock, acute respiratory distress syndrome, acute kidney injury, disseminated intravascular coagulation and so on (3).

At present, the prevalence of COVID-19 is causing fear and panic, and the society urgently needs to know the mental health status in time (4). Prior research has revealed a deep and wide range of psychosocial impacts [post-traumatic stress disorder (PTSD), depression, generalized anxiety disorder, panic disorder, and substance abuse] on people at the individual, community, and international levels during outbreaks of infection (5–7). Especially, isolation separates persons may have been in close contact with a confirmed or suspected case of coronavirus (and thus at risk for disease) from the general public. For the sake of greater public good, isolation may bring heavy economic, emotional, and psychological problems for some people. During the Middle East Respiratory Syndrome (MERS) epidemic, the prevalence of anxiety symptoms and anger in persons isolated were examined (5). According to our research, most of the studies related to this outbreak have focused on identifying the epidemiological and clinical characteristics of infected patients (8, 9), the genomic characterization of the virus (10), psychological distress among medical staff (11) and general population (12, 13). However, there are no researches investigating the psychological impact on the isolated population due to risk of infection during the COVID-19 epidemic.

The objective of this psychological impact survey was to seizure a range of responses (including depression, anxiety, and PTSD symptoms) of isolated persons in Hubei within the past month and a half of the COVID-19 outbreak to better understand their psychologic status and potential danger. This may be helpful for government agencies and healthcare professionals to protect the mental health of the pubic, particular the isolated individuals, in the face of COVID-19 outbreak expansion in China and around the world. We hypothesized that a number of respondents had moderate-to-severe depression, anxiety and PTSD symptoms, and the risk factors (the causes and conditions that lead to or increase the chance of risk accidents or enlarge the scope of loss) for the psychological impact might relate to isolation places, knowledge of the disease, knew someone to have Covid-19, and occupation.

## Methods

### Setting and Participants

We used a cross-sectional survey design and anonymous online questionnaire composed of 75 single choice and short-answer questions to evaluate the demographic characteristics, isolated places, contact history, knowledge of COVID-19 and immediate psychological response of isolated population in Hubei Province during the prevalence of COVID-19. Since the incubation period of COVID-19 is range from 1–14 days, isolated individuals who may have been in close contact with confirmed cases during the period of 14 days were isolated for 2-week in the homes or centralized quarantined spots. Each person was isolated in one single room. Isolated persons were asked not to leave their quarantined areas or have visitors, and instructed to measure their temperature twice daily. If any symptoms of COVID-19 developed (sore throat, a cough, fever, tiredness, or shortness of breath), they were to call hospital for urgent assessment. Every respondent had his or her own IP address, and at the end of the questionnaire, we would check carefully the IP address and delete the questionnaire with the same IP address. This study was approved by the Ethics Committee of First Affiliated Hospital of Jinan University (Guangzhou, China, Approval Letter: KY-2020-044), and informed, written consents were obtained from all participants.

### Survey Instrument

The psychological impact of isolation was evaluated with validated scales, including the Chinese version Patient Health Questionnaire-9 (PHQ-9), the 7-item Generalized Anxiety Disorder Scale (GAD-7), the Impact of Event Scale-Revised (IES-R), and the Simplified Coping Style Questionnaire-20 (SCSQ-20) (14). The PHQ-9 is a nine-item questionnaire, each with a Likert rating scale from 0 to 3, designed to screen for depression in primary care and other medical settings (15). The maximum score is 27. The standard cut-off score for screening to identify moderate-severe depression is 10 or above, which was established in the first study on the PHQ-9 (16). Item 9 of the PHQ-9 evaluates passive thoughts of death or self-injury within the last 2 weeks, and is often used to screen depressed patients for suicide risk (17). GAD-7 is composed of 7-item highly relevant questions with 4-point Likert scoring system from 0 to 3, which is a self-administered test to assess generalized anxiety disorder. The maximum total score is 21. In this study, the total score ≥10 points was used as the cutoff score for moderate anxiety symptoms, and the individuals with that score were categorized into the anxiety group (17). The IES-R is a self-report measure designed to assess current subjective distress resulting from a traumatic life event and is composed of 22 items, each with a Likert rating scale from 0 to 4. The maximum score is 88. The standard cut-off score for screening to identify possible PTSD symptoms is 20 (6, 18). The SCSQ-20 is a 20-item self-report questionnaire that includes two dimensions, active coping (12 items) and passive coping (8 items), each with a Likert rating scale from 0 “never” to 3 “very often.”

### Statistical Analysis

Descriptive statistics were calculated for demographic characteristics, psychological symptoms, isolated places, contact history, knowledge of COVID-19, and concern-related variables by using SPSS statistical software 19.0 (SPSS, Chicago, II, USA). The original scores of the IES-R, PHQ-9, GAD-7, active coping and passive coping were not normally distributed and so are presented as median with interquartile ranges (IQRs). For categorical variables, group proportions were calculated according to the number of respondents per response with respect to the number of total responses of a question. The nonparametric Mann–Whitney *U*-test and Kruskal–Wallis test were applied to compare the symptoms of PTSD, depression, anxiety, active coping and passive coping. The Cronbach α coefficient was calculated to evaluate the internal consistency of the responses given to the scale. Cronbach α is unreliable between 0.0 and 0.40, low reliable between 0.40 and 0.60, quite reliable between 0.60 and 0.80, and highly reliable between 0.80 and 1.00. The *P*-value is accepted as <0.05 as the statistically significant level. To identify potential risk factors for symptoms of PTSD, depression and anxiety in isolated respondents, multivariable logistic regression analysis was performed, and the odds ratios (ORs) and 95% confidence intervals (CIs) were obtained from logistic regression models, after adjustment for confounders, including sex, age, education level, marital status, occupation, isolation places, geographic location in Wuhan or not, knowledge of epidemic, knew someone to have COVID-19, active coping and passive coping. A score of ≥10 on the PHQ-9 was used to estimate the prevalence of depressive symptoms. A score of ≥10 on the GAD-7 was used to estimate the prevalence of anxiety symptoms. A score of ≥20 on the IES-R was used to estimate the prevalence of PTSD symptoms.

## Results

### Demographics and Description of Isolated Persons

This study received a total of 1,711 questionnaires from Hubei province, 396 questionnaires not filled out completely correctly were excluded, leading to inclusion of 1,315 valid questionnaires with no missing data. Demographic characteristics are presented in Table 1. Among all the isolated persons, the majority of respondents were men (59.3%), aged 26–35 years (50.8%), geographic location in Wuhan (58.5%), married (68.3%), worse educated (54.1% ≤ senior high school), self-employed (40.4%), know well of the epidemic (57.1%), isolation at home (69.3%), knew someone to have COVID-19 (63.3%).

**Table 1:** Characteristics of isolated persons who responded to the survey.

**Characteristic**	**No. (%) (*N* = 1,315)**
**Gender**	
Male	780 (59.3)
Female	535 (40.7)
**Age**	
<18	27 (2.0)
18–25	364 (27.7)
26–35	668 (50.8)
36–45	222 (16.9)
46+	34 (2.6)
**Education**	
Senior high school or below	711 (54.1)
Bachelor's degree or above	604 (45.9)
**Geographic location**	
Wuhan	769 (58.5)
Other cities in Hubei	546 (41.5)
**Occupation**	
Medical staff	62 (4.7)
Students	111 (8.4)
Self-employed	532 (40.4)
Farmers	162 (12.3)
Employed	384 (29.2)
Unemployed	64 (4.9)
**Marital status**	
Single or divorced or widowed	417 (31.7)
Married	898 (68.3)
**Knowledge of the epidemic**	
Don't know much	127 (9.7)
Know well	751 (57.1)
Very familiar with	437 (33.2)
**Isolation places**	
At home	911 (69.3)
At centralized quarantined spot	404 (30.7)
**Know someone to have COVID-19**	
Yes	832 (63.3)
No	483 (36.7)
**Relationship with infected patients**	
Man and wife	117 (14.1)
Parents	175 (21.0)
Offsprings	106 (12.7)
Brothers and sisters	97 (11.7)
Friends	331 (39.8)
Others	6 (0.7)

### Psychological Impact and Coping Style

The psychological impact of COVID-19 outbreak, measured using the IES-R scale, revealed a sample median score of 39.0, IQR (30.0–47.0). Almost 89.0% of the respondents experienced PTSD symptoms. Depression level of respondents was measured by PHQ-9 scale, revealed a sample median score of 12.0, IQR (8.0–14.0), 879 (66.8%) rated moderate-severe depression. According to PHQ-9 item 9, 515 (39.2%) were considered to be with suicide and self-injury risk. Respondents' anxiety levels, measured using the GAD-7 item scale, revealed a sample median score of 9.0, IQR (6.0–12.0). Of all respondents, 654 (49.7%) were considered to suffer from moderate-severe anxiety. The coping style of all respondents by using SCSQ-20 scale revealed a sample median score of 20.0, IQR (16.0–24.0) of active coping style, 13.0, IQR (10.0–15.0) of passive coping style. Moreover, people isolated at centralized quarantined spots had higher IES-R, PHQ-9, GAD-7, and passive coping scores, than those isolated at home. Persons who knew someone to have COVID-19 (including family and friends) had higher IES-R, active and passive coping scores. Persons who were very familiar with the epidemic had higher scores in IES-R, active coping, and passive coping. The mean IES-R, PHQ-9, GAD-7, active coping, and passive coping scores were not different for male or female (*P* > 0.05). Persons aged 36–45 years had higher passive coping scores; married and well-educated respondents had higher active and passive coping scores. All the above differences were statistically significant (*P* < 0.05; Table 2; Supplementary Table 1).

**Table 2:** Prevalence of PTSD, depressive, anxiety symptoms, and coping style according to respondents' demographics.

**Characteristic**	**No. (%) (*N* = 1,315)**	**Total score, median (IQR)**	
Prevalence			
IES-R, PTSD symptoms		39.0 (30.0–47.0)	
<20	145 (11.0)		
≥20	1,170 (89.0)		
PHQ-9, depressive symptoms		12.0 (8.0–14.0)	
<10	436 (33.2)		
≥10	879 (66.8)		
PHQ-9, depressive symptoms			
0–4 (Normal)	158 (12.0)		
5–9 (Mild)	278 (21.1)		
10–14 (Moderate)	563 (42.8)		
15–27 (Severe)	316 (24.0)		
GAD-7, anxiety symptoms		9.0 (6.0–12.0)	
<10	661 (50.3)		
≥10	654 (49.7)		
GAD-7, anxiety symptoms			
0–4 (Normal)	226 (17.2)		
5–9 (Mild)	435 (33.1)		
10–14 (Moderate)	555 (42.2)		
15–21 (Severe)	99 (7.5)		
SCSQ-20, coping styles			
Active coping		20.0 (16.0–24.0)	
Passive coping		13.0 (10.0–15.0)	
**Isolation places**	**Median (IQR)**	***P-*** **value**	***Z-*** **value**
IES-R		0.005	−2.814
Home (*n* = 911)	38.0 (28.0–47.0)		
Centralized quarantined spot (*n* = 404)	40.0 (33.0–47.8)		
PHQ-9		<0.001	−5.482
Home	11.0 (7.0–14.0)		
Centralized quarantined spot	13.0 (10.0–15.0)		
GAD-7		<0.001	−4.199
Home	9.0 (6.0–11.0)		
Centralized quarantined spot	10.0 (7.0–12.0)		
Passive coping		0.029	−2.181
Home	13.0 (10.0–15.0)		
Centralized quarantined spot	13.0 (11.0–15.0)		
**Know someone to have COVID-19**	**Median (IQR)**	***P*** **-value**	***Z-*** **value**
IES-R		<0.001	−4.724
Yes (*n* = 832)	40.0 (31.0–49.0)		
No (*n* = 483)	37.0 (28.0–44.0)		
Active coping		<0.001	−4.166
Yes	21.0 (17.0–25.0)		
No	19.0 (15.0–22.0)		
Passive coping		<0.001	−4.151
Yes	13.0 (11.0–16.0)		
No	12.0 (10.0–15.0)		
IES-R		0.022	7.659
Don't know much (*n* = 127)	36.0 (30.0–45.0)		
Know well (*n* = 751)	39.0 (31.0–45.0)		
Very familiar with (*n* = 437)	41.0 (28.0–52.0)		
Active coping		<0.001	129.678
Don't know much	18.0 (15.0–21.0)		
Know well	19.0 (16.0–22.0)		
Very familiar with	23.0 (18.0–28.0)		
Passive coping		<0.001	44.217
Don't know much	13.0 (10.0–14.0)		
Know well	12.0 (10.0–15.0)		
Very familiar with	14.0 (11.0–17.0)		

### Internal consistency

The Cronbach α for the IES-R, PHQ-9, GAD-7, SCSQ-20 active coping, SCSQ-20 passive coping, and total SCSQ-20 coping adapted in the study were 0.935, 0.847, 0.843, 0.863, 0.779, and 0.888, respectively. Thus, it can be said that all scales are reliable tools.

### Risk Factors of PTSD, Depressive, and Anxiety Symptoms

According to the results of multivariable logistic regression analysis, after controlling for other confounders including sex, age, education level, marital status, and occupation, persons who isolated at home was associated with a lower risk of PTSD, depressive and anxiety symptoms (OR = 0.61, 95% *CI*: 0.39–0.96, *P* = 0.031; OR = 0.59, 95% *CI*: 0.45–0.79, *P* < 0.001; OR = 0.68, 95% *CI*: 0.53–0.88, *P* = 0.003). Persons who knew someone to have COVID-19 were associated with severe symptoms of PTSD symptoms (OR = 1.94, 95% *CI*: 1.32–2.87, *P* = 0.001). As for coping style, higher level of passive coping style (OR = 1.23, 95% *CI*: 1.17–1.29, *P* < 0.001; OR = 1.17, 95% *CI*: 1.13–1.21, *P* < 0.001; OR = 1.13, 95% *CI*: 1.09–1.17, *P* < 0.001) was associated with severe symptoms of PTSD, depression and anxiety. Compared with unemployed, students (OR = 4.08, 95% *CI*: 1.36–12.24, *P* = 0.012; OR = 2.08, 95% *CI*: 1.04–4.17, *P* = 0.039), farmers (OR = 2.63, 95% *CI*: 1.08–6.43, *P* = 0.034; OR = 2.62, 95% *CI*: 1.37–4.99, *P* = 0.004) and employed (OR = 2.26, 95% *CI*: 1.05–4.87, *P* = 0.037; OR = 2.44, 95% *CI*: 1.36–4.37, *P* = 0.003) were associated with severe symptoms of PTSD and depression. And medical staff (OR = 2.46, 95% *CI*: 1.11–5.45, *P* = 0.026) and self-employed (OR = 2.04, 95% *CI*: 1.15–3.60, *P* = 0.014) were also associated with severe symptoms of depressive symptoms. Additionally, men were associated with severe symptoms of depressive symptoms than women (OR = 1.41, 95% *CI*: 1.09–1.81, *P* = 0.009). Compared with didn't know much of the epidemic, persons who knew well the epidemic were associated with severe depressive symptoms (OR = 1.63, 95% *CI*: 1.07–2.47, *P* = 0.022). Persons who were geographic location in Wuhan were associated with a lower risk of anxiety symptoms (OR = 0.77, 95% *CI*: 0.61–0.98, *P* = 0.031). Compared with married, single or divorced or widowed was associated with a lower risk of anxiety symptoms (OR = 0.74, 95% *CI*: 0.57–0.97, *P* = 0.030) (Table 3).

**Table 3:** Results of multivariate logistic regression analyses.

**Variable**	**No. of severe cases/no. of total cases (%)**	**OR (95% *CI*)**	***P-*value**
**IES-R, PTSD symptoms**			
Isolation places			
Home	797/911 (87.5)	0.61 (0.39–0.96)	0.031
Centralized quarantined	373/404 (92.3)	1 [Reference]	NA
spot			
Know someone to have COVID-19			
Yes	762/832 (91.6)	1.94 (1.32–2.87)	0.001
No	408/483 (84.5)	1 [Reference]	NA
Passive coping	NA	1.23 (1.17–1.29)	<0.001
Occupation			
Medical staff	56/62 (90.3)	2.36 (0.76–7.38)	0.140
Students	104/111 (93.7)	4.08 (1.36–12.24)	0.012
Self-employed	464/532 (87.2)	1.78 (0.86–3.69)	0.120
Farmers	149/162 (92.0)	2.63 (1.08–6.43)	0.034
Employed	347/384 (90.4)	2.26 (1.05–4.87)	0.037
Unemployed	50/64 (78.1)	1 [Reference]	NA
**PHQ-9, depressive symptoms**			
Isolation places			
Home	574/911 (63.0)	0.59 (0.45–0.79)	<0.001
Centralized quarantined	305/404 (75.5)	1 [Reference]	NA
spot			
Passive coping	NA	1.17 (1.13–1.21)	<0.001
Gender			
Male	541/780 (69.3)	1.41 (1.09–1.81)	0.009
Female	338/535 (63.2)	1 [Reference]	NA
Occupation			
Medical staff	43/62 (69.3)	2.46 (1.11–5.45)	0.026
Students	73/111 (65.8)	2.08 (1.04–4.17)	0.039
Self-employed	347/532 (65.2)	2.04 (1.15–3.60)	0.014
Farmers	117/162 (72.2)	2.62 (1.37–4.99)	0.004
Employed	276/384 (71.9)	2.44 (1.36–4.37)	0.003
Unemployed	32/64 (50.0)	1 [Reference]	NA
Knowledge of the epidemic			
Don't know much	76/127 (59.8)	1 [Reference]	NA
Know well	522/751 (69.5)	1.63 (1.07–2.47)	0.022
Very familiar with	281/437 (64.3)	1.01 (0.64–1.58)	0.979
**GAD-7, Anxiety symptoms**			
Isolation places			
Home	422/911 (46.3)	0.68 (0.53–0.88)	0.003
Centralized quarantined	232/404 (57.4)	1 [Reference]	NA
spot			
Geographic location			
Wuhan	358/769 (46.5)	0.77 (0.61–0.98)	0.031
Other cities in Hubei	296/546 (54.2)	1 [Reference]	NA
Active coping	NA	1.04 (1.01–1.06)	0.002
Passive coping	NA	1.13 (1.09–1.17)	<0.001
Marital status			
Single or divorced or	186/417 (44.6)	0.74 (0.57–0.97)	0.030
widowed			
Married	468/898 (52.1)	1 [Reference]	NA

## Discussion

The median incubation period of COVID-19 is an average of 5–6 days (1–14 days), so symptoms typically occur a minimum of 1 day or a maximum of 14 days after exposure to the coronavirus (19). Thus, people who have been in close contact should be monitored closely for at least 14 days for occurrence of symptoms. Our results show that a substantial proportion of isolated persons experienced psychological problems, as evidenced by the proportion that display symptoms of depression (66.9%), anxiety (49.8%), and PTSD (89.0%) symptoms as measured by validated scales. With respect to the recent global outbreak of COVID-19, considerable time has been spent discussing the specifics of isolation and how to promote adherence to infection control measures. Little, if any, analysis has focused on the effect of isolation on the psychological health of the isolated person. This knowledge is critical if modern isolation is to be an effective disease-containment strategy. To our knowledge, a consideration of the adverse psychological effects of isolated populations in Hubei has not previously been systematically endeavored since the outbreak of COVID-19.

The prevalence of depression, anxiety and PTSD symptoms in our study population was higher than that reported during the outbreak of SARS (6, 20), MERS (5), and Ebola (21). The current COVID-19 outbreak is different to the prior SARS or MERS, which is creating a confused and rapidly evolving situation. It has stronger human-to-human transmission capability, and it can even be transmitted through asymptomatic individuals, while the health authorities had insufficient preparedness to address the outbreaks, thus there is greater unpredictability and can cause more panic. Despite much higher case-fatality rate (CFRs) for SARS and MERS, COVID-19 has led to more total deaths due to the large number of cases, the CFR was as much as 49.0% among critical cases (the overall CFR was 2.3%) (22), nevertheless, no proper treatment or vaccine is available for the epidemic. To reduce potential transmission from exposed persons before symptoms occur so as to lower the risk of further disease transmission, who may have been in close contact with confirmed or suspected cases during the period of 14 days were isolated for 2-week in the homes or centralized quarantined spots. This takes a considerable toll on the person. Those in isolation might experience boredom, loneliness, anger, guilt about the effects of contagion, quarantine, and stigma on their families and friends (4), could lead to mental distress, persistent anxiety and depression, panic attacks, psychomotor excitement, psychotic symptoms, delirium, and even suicidality as reported in the early phase of the SARS outbreak (23). We identified for increased depression, anxiety and PTSD symptoms in the quarantined persons at the centralized quarantined spot and those with infected patients around, which brought benefits and challenges to the prevention and control of COVID-19. It is noteworthy that unemployed persons were less at risk of PTSD and depression, probably because they didn't worry about delays in work time and subsequent deprivation of their anticipated income due to virus exposure in public transportation (24). While, people who knew well about the epidemic was associated with a higher risk of depression, probably because they tend to obtain a large amount of information that can easily trigger stress (25).

A score of ≥20 on the IES-R was used to estimate the prevalence of PTSD symptoms based on the study of journalists working in war zones (18) and SARS control and psychological effects of quarantine (6). While other cutoff points may have been used to estimate the prevalence of PTSD symptoms (26, 27), what we identified was increased risk factors for PTSD symptoms, rather than the absolute prevalence of PTSD in our study participants, which is the important findings of this study. In this survey, the presence of PTSD symptoms was up to 89% and highly positively associated with one's depression, anxiety, and passive coping styles. According to Diagnostic and Statistical Manual of Mental Disorders, 5th Edition (DSM-5), the diagnostic criteria for PTSD are as follows, the individual has been exposed to a traumatic event that combines two factors: A1 the individual has witnessed or encountered one or more physical deaths involving himself or others, or has been threatened with death, or has been severely injured, or has been threatened with physical integrity; A2 the individual's reactions include intense fear, helplessness, or panic. People who knew someone to have COVID-19 are related to the increase of PTSD symptoms, probably because of high self-awareness of their health, more worries about relatives and friends, and more exposure to the threat of death (28). Progression of depression and anxiety symptoms experienced in the early stages of natural disaster can be prevented by early mental health care (29). However, these symptoms evolve into long-term PTSD without early intervention. This study also noted the trend toward increasing symptoms of both PTSD and depression as the passive coping of the respondent. Active coping styles reduce psychological problems, while passive coping styles increase mental issues (30). Isolated persons with risk factors for either anxiety, depression or PTSD symptoms may benefit from increased support from public health officials. Relief materials must be provided in a timely manner during the period of isolation. Accurate information about the symptoms of the disease should be publicly available, and psychological support is needed for patients who have persistent symptoms even after the isolation is removed. Any financial loss should be recognized and appropriately supported. Psychological support is necessary for people with a history of mental illness because they are more likely to experience mental symptoms. Medical management plan should be provided for patients with persistent symptoms.

Although isolated persons underwent symptoms suggestive of depression, anxiety and PTSD, the scales used to measure these symptoms are not sufficient to confirm these diagnoses. Structured diagnostic interviews are required to confirm the diagnoses of depression, anxiety and PTSD. This was not possible because the survey was anonymous. And it's worth mentioning that we found 515 (39.2%) isolated persons were considered to be with suicide and self-injury risk through item 9 of PHQ-9. Though previous study suggested that item 9 of the PHQ-9 was an insufficient assessment tool for suicide risk and suicide ideation (17), the possibility cannot be completely ignored.

We investigated the psychological status and coping styles of isolated populations of the COVID-19 epidemic from 1,315 respondents in Hubei province. The sample size was larger than that of most related studies. Although Hubei province is the birthplace of the epidemic, the isolated populations in other provinces may have similar psychological conditions because of COVID-19. In addition, we can make a comparative study on the psychological status of the isolated populations in Hubei province before and after the blockade in the future. While, there are several limitations in this study. First, the actual number of isolated people is low than the total number of persons who were placed into isolation (the exact number is unknown), so it may not represent of the whole group of isolated persons. However, due to lack of funding, confidentiality of public health records and an overloaded public health response system, the sampling of this studies limited. Second, a self-selection effect may have occurred with those persons who were experiencing the greatest or least levels of distress responding to the survey. Third, respondents need to use a computer or smartphone to respond, suggesting that they may be more educated and socio-economic than the quarantined population as a whole. Fourthly, we didn't indicate the means of communication during isolation with family as with a medical staff, as well as information about the psychiatric history of isolated person. Fifthly, all measures used in this study were based on self-reports, which were very subjective. In addition, the age range of the included participants in this study was mostly from 26 to 35 years old who is very young and is vulnerable to psychological problems, which may bias the conclusions. Finally, we just did a cross-sectional study, and we didn't follow up with people who were quarantined.

## Conclusion

Our data show that isolation can result inconsiderable psychological distress in the forms of depressive, anxiety, and PTSD symptoms. Public health officials, infectious diseases physicians, psychiatrists and psychologists need to be aware of this issue. They must strive to identify the factors that affect the success of isolation and infection control measures in disease control and community rehabilitation, and must be prepared to provide additional support to those who are at greater risk of adverse psychological and social consequences of isolation.

## Data Availability Statement

The raw data supporting the conclusions of this article will be made available by the authors, without undue reservation.

## Ethics Statement

This study was approved by the Ethics Committee of First Affiliated Hospital of Jinan University (Guangzhou, China, Approval Letter: KY-2020-044), and informed, written consents were obtained from all participants.

## Author Contributions

YW and LH designed the study. JG, GC, ZQ, SZ, TS, YP, and JW contributed to data acquisition. GC and YW contributed to data analysis. JG and GC wrote the manuscript. YW and LH revised the manuscript. All authors contributed and approved the final manuscript.

## Conflict of Interest

The authors declare that the research was conducted in the absence of any commercial or financial relationships that could be construed as a potential conflict of interest.

## Publisher's Note

All claims expressed in this article are solely those of the authors and do not necessarily represent those of their affiliated organizations, or those of the publisher, the editors and the reviewers. Any product that may be evaluated in this article, or claim that may be made by its manufacturer, is not guaranteed or endorsed by the publisher.
